# Plasma calcium concentration during detoxification predicts neural cue-reactivity and craving during early abstinence in alcohol-dependent patients

**DOI:** 10.1007/s00406-021-01240-4

**Published:** 2021-02-25

**Authors:** Patrick Bach, Rilana Schuster, Anne Koopmann, Sabine Vollstaedt-Klein, Rainer Spanagel, Falk Kiefer

**Affiliations:** 1 Department of Addictive Behavior and Addiction Medicine, Medical Faculty Mannheim, Heidelberg University, Central Institute of Mental Health, Square J5, 68159 Mannheim, Germany; 2 Feuerlein Center on Translational Addiction Medicine, Heidelberg University, Heidelberg, Germany; 3 Medical Faculty Mannheim, Heidelberg University, Institute of Psychopharmacology, Central Institute of Mental Health, Heidelberg, Germany; 4 Spinal Cord Injury Center, Heidelberg University Hospital, Schlierbacher Landstraße 200 a, 69118 Heidelberg, Germany

**Keywords:** Alcohol dependence, Calcium, Craving, FMRI, BOLD response

## Abstract

Recent studies on the pathophysiology of alcohol dependence suggest a link between peripheral calcium concentrations and alcohol craving. Here, we investigated the association between plasma calcium concentration, cue-induced brain activation, and alcohol craving. Plasma calcium concentrations were measured at the onset of inpatient detoxification in a sample of *N* = 115 alcohol-dependent patients. Alcohol cue-reactivity was assessed during early abstinence (mean 11.1 days) using a functional magnetic resonance imaging (fMRI) alcohol cue-reactivity task. Multiple regression analyses and bivariate correlations between plasma calcium concentrations, clinical craving measures and neural alcohol cue-reactivity (CR) were tested. Results show a significant negative correlation between plasma calcium concentrations and compulsive alcohol craving. Higher calcium levels predicted higher alcohol cue-induced brain response in a cluster of frontal brain areas, including the dorsolateral prefrontal cortex (dlPFC), the anterior prefrontal cortex (alPFC), and the inferior (IFG) and middle frontal gyri (MFG). In addition, functional brain activation in those areas correlated negatively with craving for alcohol during fMRI. Higher peripheral calcium concentrations during withdrawal predicted increased alcohol cue-induced brain activation in frontal brain areas, which are associated with craving inhibition and cognitive control functions. This might indicate that higher plasma calcium concentrations at onset of detoxification could modulate craving inhibition during early abstinence.

**Trial registration number**: DRKS00003388; date of registration: 14.12.2011.

## Introduction

The potential role of calcium metabolism as a target for treatment of alcohol dependence (AD) was rather undervalued for a long period until Spanagel and colleagues [23] published translational data, suggesting that calcium is the active component of one of the worldwide most frequently used anti-craving medication acamprosate (calcium-bis N-acetylhomotaurinate). The study showed higher plasma calcium levels under acamprosate treatment compared to placebo and in addition that higher plasma calcium levels were associated with increased time to relapse and higher cumulative abstinence periods. While the effect of calcium in relation to acamprosate effects is still under debate [7, 13, 24], the effect of calcium balance in patients with AD was recently investigated. Plasma concentrations of calcium, calcitonin, vitamin D, and parathyroid hormone [19] were investigated in alcohol-dependent subjects. Our group found a negative correlation between plasma calcium concentration and alcohol craving, a negative correlation between plasma calcium concentration and breath alcohol concentration, a decreased calcitonin concentration in a “heavily drinking” subgroup, and a significantly reduced vitamin D plasma concentration in all alcohol-dependent individuals. While the study pointed towards a negative association between plasma calcium levels and alcohol craving [19], the neurobiological basis of this association remained to be elucidated. Given the tight regulation of plasma calcium levels and the fact that calcium is an essential messenger for intracellular and nuclear signaling [3] and as such critical for the regulation of brain function, it is surprising that peripheral calcium levels should influence brain function. However, since serum and brain interstitial fluids maintain equilibrium for ion concentrations via passive diffusion, the amount of peripheral calcium could potentially affect brain functions. Preclinical studies indicated that intracellular calcium increases in neurons, especially in astrocytes, are involved in vascular signals promoting vasodilation and thereby producing a BOLD signal [10]. Schulz and colleagues [18] compared fMRI and fiber-optic recordings of fluorescent calcium indicator signals in the somatosensory cortex of rats. They found that BOLD responses could be predicted from simultaneously recorded fiber-optic signals. In addition, Duong and colleagues [5] showed spatial overlap between measures of synaptic activity, BOLD response, and cerebral blood flow. A recent study indicated that increased neuronal activation, upon sensory stimulation, is correlated to astrocytic calcium signals and BOLD fMRI signal [28]. Previous optical imaging studies have also indicated that astrocytic calcium signaling may be involved in neurovascular vessel dilation contributing to a positive BOLD fMRI signal [7, 12, 29]. In an epidemiological study, it was shown that calcium in drinking water has protective effects on cognitive decline in older persons (age > 65 years), depending on the given dosage [6]. This effect was measurable at 86 mg/l calcium in the drinking water, but in higher calcium doses, the effect disappeared. Furthermore, in patients with depression, recent published data showed an association between higher serum calcium concentrations and neuropsychological processing, executive function, and global assessment of functioning [20], supporting the association between plasma calcium and cognitive functioning. Based on the preclinical evidence linking calcium to BOLD signal changes and the findings of a negative association between plasma calcium and craving in a previous study of our workgroup [19, 23], we expected to replicate the negative association between plasma calcium levels and alcohol craving and hypothesized to detect a positive association between plasma calcium levels and alcohol-cue-induced brain response in brain areas engaged in craving inhibition and cognitive control.

### Experimental procedures

The study adhered to the Declaration of Helsinki and was approved by the local ethics committee of the University Medical Center in Mannheim, Germany, and was registered at the national study registry (DRKS00003388).

### Participants

We investigated the association between plasma calcium concentrations and alcohol cue-induced neural response in 115 recently detoxified patients with alcohol dependence (AD) (32 females, 27.8%), who were recruited from the Department of Addictive Behavior and Addiction Medicine at the Central Institute for Mental Health (Mannheim, Germany) between 2008 and 2011 in the framework of a larger study that expanded from 2008 to 2015. The current study reports data from a cohort, which was recruited between 2008 and 2011 and consisted of 133 alcohol-dependent patients of whom 115 provided complete fMRI datasets and plasma blood samples for calcium measurement (9 patients dropped out due to missing plasma blood samples and 9 patients dropped out due to movement artefacts during fMRI), Healthy controls were also recruited in the framework of the project, but no plasma samples were available for this cohort, and hence, these participants could not be included in current analyses. All patients were screened prior to the experiment and written informed consent was obtained from all participants in accordance with the Declaration of Helsinki. Inclusion criteria were: (i) ages between 18 and 65 years; (ii) diagnosis of an AD according to the diagnostic statistical manual of mental disorders (DSM-IV). Exclusion criteria were: (i) comorbid Axis-I disorders (other than alcohol dependence and nicotine dependence) in the last 12 months, (ii) current psychoactive or anticonvulsive medication, (iii) comorbid severe physical or neurological disease (including any liver function deficits, signs for liver cirrhosis, osteoporosis, thyroidal dysfunction, or renal dysfunction or insufficiency (GFR < 90 ml/min), (iv) positive drug screening, (v) present contraindications for performing an MRI scan (e.g., tattoos, metal implants, pregnancy, pacemakers), (vi) anti-craving medication the last 14 days before scanning, and (vii) a history of severe withdrawal symptoms (i.e., any past delirium or seizures during withdrawal treatment or any past intensive-care treatment during alcohol withdrawal).

### Psychometric assessments

Patients were surveyed for demographical and clinical data. All participants were asked to complete a series of questionnaires at baseline before the fMRI scan was performed, including the obsessive compulsive drinking scale (OCDS) [2], the alcohol dependence scale (ADS) [21], and the Beck’s depression inventory (BDI-II) [9, 14].

### Plasma calcium measurements

Plasma calcium concentration was determined in blood plasma of patients at the day of admission to inpatient detoxification treatment. Laboratory testing was conducted immediately after acquisition of the blood sample. In vitro test for the quantitative determination of calcium in human plasma was conducted on a COBAS INTEGRA system (Roche Diagnostics, Rotkreuz, Switzerland). The measuring range accounts for plasma levels between 0.20 and 5.0 mmol/L (0.8–20.1 mg/dL).

### fMRI alcohol cue-reactivity task

The mean abstinence duration at the time point of the scanner session was 11.1 (SD = 5.87) days. We applied an established alcohol picture cue-reactivity task that was repeatedly validated in previous studies, e.g., [27]. In this task, series of 5 alcohol-related or neutral pictures were presented to participants via MRI compatible googles (MRI Audio/Video Systems, Resonance Technology Inc., Los Angeles, CA, USA) in a pseudo-randomized order while in the scanner. In total, 12 blocks of alcohol pictures (5 pictures per block) and 9 blocks of neutral pictures (5 pictures per block) were displayed. Each picture was presented for 4 s. In between successive blocks, participants were asked to report their current craving for alcohol on a visual analogue scale from 0 (“no craving at all”) to 100 (“very intense craving”). Participants were asked to press a button, while the scale from 0 to 100 was running to rate their craving. The task took approximately 12 min to complete in its entirety. Data presentation and recording was monitored using the Presentation^®^ software (Version 16.0, Neurobehavioral Systems Inc., Albany, CA, USA).

### fMRI acquisition, pre-processing, and statistical analyses

Brain response was measured using a Siemens MAGNETOM 3 T whole-body-tomography (MAGNETOM Trio, TIM technology, Siemens, Erlangen, Germany). A total of 305 T2*-weighted echo-planar images (EPI) were acquired per participant during the alcohol cue-reactivity task with standardized imaging parameters (TR = 2.41 s, TE = 25 ms, flip angle = 80°, 42 slices, slice thickness = 2 mm, 1-mm gap, voxel dimensions 3 × 3 × 3 mm^3^, FOV = 192 × 192 mm^2^, and 64 × 64 in-plane resolution). The first five T2*-weighted EPI images were dropped from successive analyses to avoid bias due to magnetic saturation effects. The remaining 300 images were pre-processed according to standard procedures implemented in the statistical parametric mapping software for MATLAB (SPM, Wellcome Department of Cognitive Neurology, London, UK) version 5 [i.e., spatial realignment, movement correction, normalization to a standard a MNI (Montreal Neurological Institute, Quebec, Canada) EPI template and smoothing using an isotropic Gaussian kernel for group analysis (8 mm Full Width at Half Maximum)]. First-level statistics were computed for every participant, modelling the different experimental conditions (alcohol pictures, neutral pictures, and rating phase) in a generalized linear model. Resulting contrast images (“alcohol—neutral”) were ascribed in second-level analyses using SPM8. Whole brain one-sample *t* tests were applied to evaluate the main effects of alcohol and neutral cues on brain response. Associations between whole brain alcohol cue-induced activation and plasma calcium levels were investigated using a multiple regression model as implemented in SPM8 with calcium as covariate of interest.

To control for multiple comparisons, a combined voxel-wise and cluster-extent-threshold, corresponding to a family-wise error (FWE) rate of *p*_FWE_ < 0.05, was determined using AlphaSim as implemented in the NeuroElf toolbox (www.neuroelf.net) for Matlab. For a pre-set voxel-wise threshold of *p* < 0.001, the AlphaSim procedure determined a cluster-extent-threshold of 63 voxels (10.000 Monte Carlo simulations, estimated smoothness based on the residual images was *x* = 14.33 mm, *y* = 14.61 mm, and *z* = 12.58 mm).

**Fig. 1:**
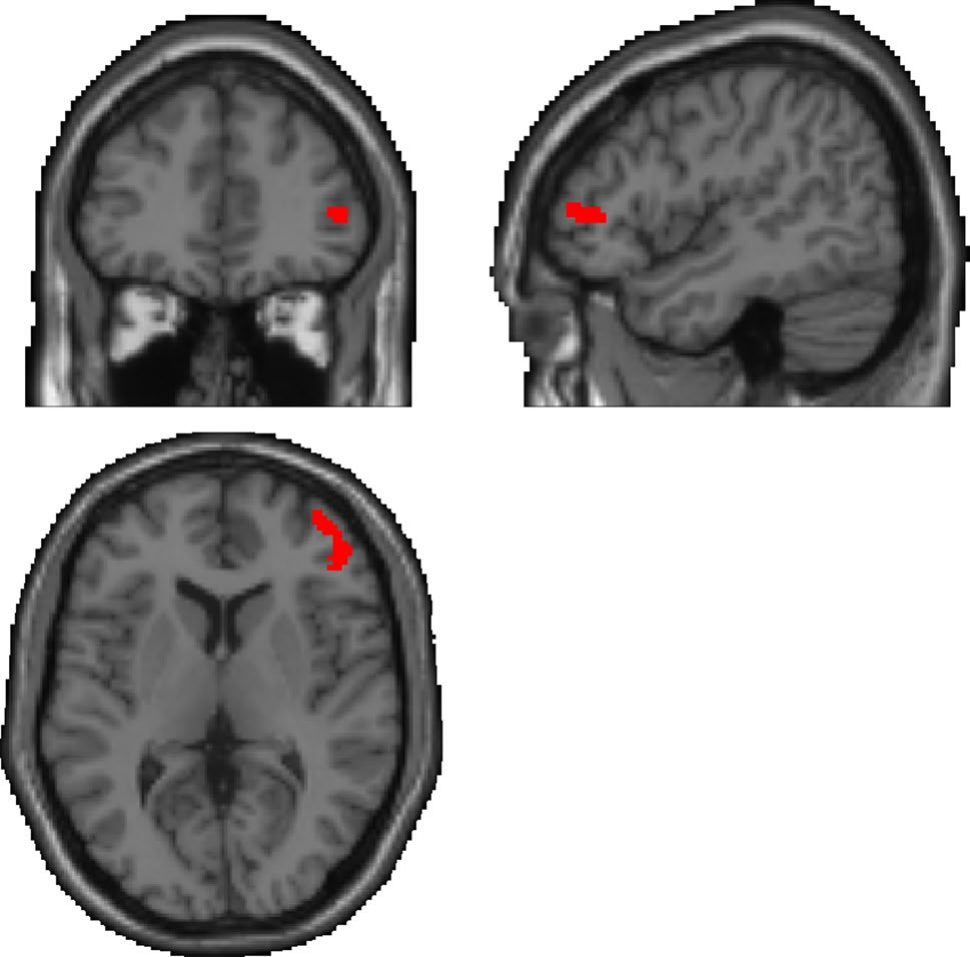
Depiction of the functional brain mask (217 voxels in total) used to extract the mean functional activation derived from computing the intersection between brain clusters demonstrating a significant positive correlation with plasma calcium scores and an anatomical mask of the right frontal lobe from the Automated Anatomical Labeling atlas (aal)

Complementing the whole brain analyses, associations between neural activation and clinical variables were investigated by computing the mean functional activation (contrast “alcohol—neutral”) for the cluster of frontal brain regions demonstrating significant associations between calcium levels and BOLD response in the multiple regression analyses. We extracted functional brain activation from the frontal activation cluster surpassing the pre-set combined voxel-wise and cluster-extent-threshold that comprised the middle (MFG) and inferior frontal gyrus (IFG), anterior prefrontal cortex (alPFC), and dorsolateral prefrontal cortex (dlPFC) (see Fig. 1). To reduce the risk to consider activation outside the frontal lobe when extracting the mean functional brain response, we computed the intersection of an anatomical mask from the Automated Anatomical Labeling atlas (aal) for the right frontal lobe region and the functional activation cluster, so that the resulting masks match anatomical borders. The resulting brain mask consisted of 217 voxels (see Fig. 1). The mean functional activation was extracted from these areas using a proprietary SPM-toolbox, which was developed in our department and has been validated through previous studies [17].

### Statistical analyses of demographical and clinical data

Data on patients’ demographics, clinical data, plasma calcium concentration and questionnaire data, as well as associations between extracted mean functional brain activation and clinical data were analysed using the Statistical Package for the Social Sciences (IBM SPSS) version 24.0. Pearson bivariate correlations were performed to investigate associations between plasma calcium levels and clinical craving measurements. The significance for all analyses was set to an alpha level of *p* < 0.05.

## Results

### Sample characteristics and cue-induced craving during fMRI

Demographical data and clinical data for all participants are shown in Table 1. The mean plasma calcium concentration was 2.4 mmol/l (± 0.10). Analyses of craving values during fMRI show that subjective craving (VAS) in response to the presentation of pictures of alcohol stimuli (Mean_Alc_ = 9.0, SD_Alc_ = 14.0) was significantly higher compared to craving during presentation of neutral picture stimuli (Mean_Neut_ = 3.2, SD_Neut_ = 7.5, *t*_(114)_ = 5.788, *p* < 0.001).

**Table 1:** Sample characteristics

Patients with alcohol dependence (*N* = 115)	Mean (SD)
Sex (male/female)	83/32
Age (years)	45.6 (9.78)
Plasma calcium (mmol/l)	2.4 (0.10)
Ethanol consumption (sum of last 90 days in gram)	10,072.8 (9593.51)
Number of detoxifications (lifetime)	2.5 (4.81)
Abstinence prior to scanning (days)	11.1 (5.87)
OCDS (obsession subscale)	5.0 (3.38)
OCDS (compulsion subscale)	9.98 (3.72)
Smoker (yes/no)	82/33
Cigarettes/day (smokers only)	1.5 (1.0)
Craving for alcohol (mean over trials)	8.9 (13.97)
FTND (total score)	5.1 (2.78)
ADS (total score)	15.8 (6.63)
BDI (total score)	10.8 (8.50)

*ADS* alcohol dependence scale, *BDI* Beck depression inventory, *FTND* Fagerstroem test for nicotine dependence, *OCDS* obsessive–compulsive drinking scale; calcium normal range [2.15–2.55 mmol/l]

### Association between plasma calcium concentrations and alcohol cue-induced craving during fMRI

We found a significant negative correlation between plasma calcium concentrations and OCDS compulsivity subscale (*r* = − 0.165, *p* = 0.042). No significant associations were found with the OCDS obsession subscale, the OCDS total score, the ADS, or the BDI scores (*p* > 0.05). The significant results do not survive correction for multiple comparisons using a Bonferroni correction (i.e., alpha 0.05/5 = 0.01).

### Alcohol cue-induced brain response

Alcohol pictures relative to neutral pictures (contrast “alcohol—neutral”) induced brain activation in a large cluster encompassing frontal cortical areas, occipital cortical areas, and mesolimbic regions like the putamen, caudate, and amygdala (see Table 2a). Contrasting neutral pictures to alcohol pictures (contrast “neutral—alcohol”) revealed significant activation in the cuneus, the lingual gyrus, and temporo-occipital regions (see Table 2b).

**Table 2:** Alcohol and neutral cue-induced brain activation (contrast “alcohol—neutral” and “neutral—alcohol”, *n* = 115, combined voxel-wise [*p* < .001] and cluster-extent-threshold [*k* > 63 voxel], corresponding to *p*_FWE_ < .05)

Side	Lobe	Brain areas	Cluster size	MNI coordinates (*x*, *y*, *z*)			*t*_max_
a) Contrast “alcohol—neutral”							
L and R	Occipital and Temporal	Superior, middle and inferior occipital gyrus, cerebellum, lingual gyrus, fusiform gyrus, calcarine. Middle and inferior temporal gyrus	11,829	− 26	− 98	− 6	12.31
L and R	Frontal	Superior, middle and inferior frontal cortex, anterior cingulum, hippocampus, cerebellum, putamen, insula, pallidum, thalamus, parahippocampal gyrus, caudate, amygdala	5990	− 28	16	− 12	6.54
R		Precuneus, middle and posterior cingulum	1146	− 6	− 52	24	5.94
L	Occipital and parietal	Superior and inferior parietal gyrus, middle occipital gyrus, angular gyrus	1338				5.74
R	Frontal	Putamen, insula, parahippocampal gyrus, inferior frontal operculum. posterior orbitofrontal cortex, amygdala, caudate	761	28	20	− 14	5.56
R	Parietal	Angular gyrus, inferior parietal gyrus	327	52	− 6	38	4.95
R	Frontal	Superior, middle and inferior frontal gyrus	305	38	54	− 2	4.74
L	Frontal	Middle frontal gyrus, precentral and postcentral gyrus	726	− 50	0	42	4.62
L	Temporal	Middle and inferior frontal gyrus	517	− 64	− 12	− 16	4.52
L and R	Frontal	Superior frontal gyrus, supplementary motor area	786	0	18	68	4.39

b) Contrast “neutral—alcohol”							
L and R		Cuneus, lingual gyrus, calcarine	580	14	− 82	8	5.56
L	Temporal, Occipital	Middle and inferior temporal gyrus, middle and inferior occipital gyrus	214	− 58	− 66	− 4	5.15
R	Temporal	Middle and inferior temporal gyrus	344	54	− 64	− 2	5.12
L	Occipital	Middle occipital gyrus	63	− 40	− 82	32	4.38
R	Temporal	Superior temporal gyrus, rolandic operculum	269	52	− 32	26	3.98

Contrast “alcohol—neutral” and “neutral—alcohol”, *n* = 115, combined voxel-wise [*p* < .001] and cluster-extent-threshold [*k* > 63 voxel], corresponding to *p*_FWE_ < .05

### Association between plasma calcium concentrations, fMRI, and craving

**Fig. 2:**
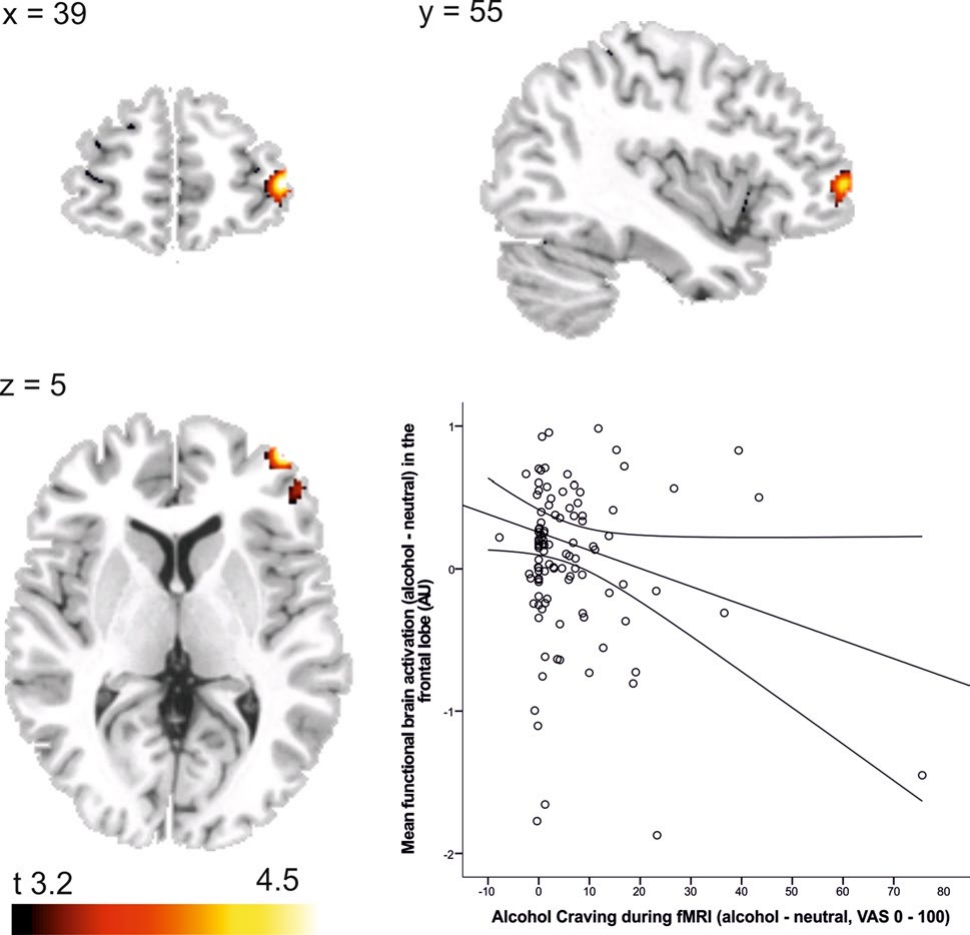
**a** Brain areas in which that show a significant positive association between alcohol cue-induced brain response and plasma calcium concentration (contrast “alcohol—neutral”, *n* = 115, combined voxel-wise threshold *p* < .001 and cluster-extent-threshold ≥ 63 voxels, corresponding to *p*_*FWE*_ < .05). **b** Scatterplot displaying the negative association between mean functional brain activation extracted from the frontal activation cluster and subjective craving during the fMRI alcohol cue-reactivity task (*r* = − 0.177, *p* = 0.029, *n* = 115)

Multiple regression analyses revealed a significant positive association between plasma calcium concentrations and alcohol-induced brain response (contrast “alcohol—neutral”) in a network of frontal brain areas that include parts of the middle frontal gyrus (MFG), inferior frontal gyrus (IFG), and parts of the antero-lateral prefrontal cortex (alPFC) (BA10) and dorsolateral prefrontal cortex (dlPFC) (BA9), as displayed in Table 3. There was no significant negative correlation between plasma calcium levels and the cue-induced blood oxygenation level-dependent (BOLD) response (*p* > 0.05). Further analyses including the mean functional brain activation (contrast “alcohol—neutral”) extracted from the frontal brain cluster that included the dlPFC, the alPFC, and the inferior and middle frontal gyri showed a negative association with subjective alcohol cue-induced craving during the fMRI session (as measured using a VAS) (*r* = − 0.177, *p* = 0.029) (Fig. 2).

**Table 3:** Positive correlation between plasma calcium levels and alcohol cue-induced brain activation

Side	Lobe	Brain areas	Cluster size	MNI coordinates (*x*, *y*, *z*)			*t*_max_
R	Frontal	Middle and inferior frontal gyrus, anterior prefrontal cortex (BA10), dorsolateral prefrontal cortex (BA9)	198	42	55	2	4.38

Contrast “alcohol—neutral”; *n* = 115; combined voxel-wise [*p* < .001] and cluster-extent-threshold [*k* > 63 voxel], corresponding to *p*_FWE_ < .05

## Discussion

Current results show that higher plasma calcium levels at admission to withdrawal treatment predicted higher alcohol cue-induced brain activation in a cluster of frontal brain areas, including the alPFC and dlPFC, which in turn showed a negative association with alcohol cue-induced craving during the fMRI session. Additionally, plasma calcium values were negatively correlated with the magnitude of subjective alcohol craving.

The association between plasma calcium concentrations and alcohol craving was established recently in a sample of alcohol-dependent patients on day 1 of detoxification [19]. Findings demonstrated a negative association between calcium concentrations and craving in these patients, such that patients with lower peripheral calcium concentration reported more alcohol craving. Current findings replicate these data by showing a negative correlation between plasma calcium levels at admission to withdrawal treatment and the OCDS obsession subscale.

To date, there are no data on the neurobiological basis of the negative association between plasma calcium levels and alcohol craving. The current study is the first to demonstrate a significant positive association between higher plasma calcium levels and alcohol cue-induced brain activation in a network of frontal brain areas, including the MFG, IFG, the alPFC (BA10), and dlPFC (BA9). While these results await replication by independent studies, preclinical data support the plausibility of the association between calcium levels and magnitude of the brain BOLD signal, e.g., by demonstrating that increased neuronal activation was correlated to astrocytic calcium signals and the BOLD fMRI signal [28] and by showing that astrocytic calcium signaling may be involved in neurovascular vessel dilation, which contributes to a positive BOLD fMRI signal [7, 12, 29].

Our data also showed a negative association between alcohol cue-induced brain activation and subjective craving during the scanning session. This is in line with previous findings that neural activation in the inferior frontal gyrus is related to craving inhibition (see [26] for review) and that alcohol cue-induced activation in the dlPFC is negatively associated with subjective alcohol craving [30]. The role of the dlPFC in craving inhibition has also been highlighted by studies investigating smoking cues [8]. Additionally, studies have found elevated dorsolateral prefrontal cortex activation in alcohol-dependent patients performing tasks that demand executive control functions [4].

Our findings are compatible with the notion that higher plasma calcium concentrations at onset of detoxification might modulate subjective alcohol craving via effects on the magnitude of alcohol cue-induced activation in craving-inhibiting prefrontal brain areas. These results are also compatible with the previous findings, which showed that acamprosate, as well as calcium chloride, improved cognitive control functions, while the sodium salt version of N-acetylhomotaurinate was not able to enhance the cognitive deficits [16].

Taken together, we could show that peripheral calcium levels were positively associated with the extent of brain activation in areas associated with craving inhibition and negatively associated with the extent of subjective alcohol craving. Even if the pathophysiology of this association remains to be clarified, we now present first evidence that calcium’s effects on craving might be mediated via its effects on brain activation. Future studies are needed to validate the role of calcium in craving modulation and to further elucidate the neurobiological basis of the observed effects.

### Limitations

Even though the current sample consisted of *N* = 115 participants and provides sufficient power to detect small, medium, and large effects of plasma calcium levels on neural brain response and clinical variables, very small effects might have remained undetected. However, the clinical importance of such very small effects might be limited. It is an ongoing debate, as to what extent calcium levels should be corrected considering albumin levels [25]. While this could be regarded as essential, when considering samples with clinical manifest somatic comorbidities, such as pancreatitis or hepatitis, several studies indicated that this is not mandatory, if calcium values stay in normal range and patients do not present with relevant somatic comorbidities, as is the case in the presented dataset [1, 11, 15, 22]. Furthermore, no extreme calcium values were detected in the current sample, reducing risk of bias. It should be noted that the correlation analyses between calcium levels and clinical scales (i.e., OCDS, ADS, and BDI) did not survive stringent correction for multiple comparisons using a Bonferroni correction and hence should be interpreted cautiously, even though the results of an independent study by Schuster et al. (2017) could show similar associations between calcium levels and craving. In addition, the time gap between the measurement of calcium levels and the fMRI signal is a relevant limitation of the current study. Currently, there are little-to-no data on the course of calcium levels during withdrawal treatment and early abstinence and future studies are needed to determine potential dynamics of calcium levels over withdrawal and abstinence. Still, unpublished data from a clinical sample of AD patients treated at the Department of Addictive Behavior and Addiction Medicine at the Central Institute for Mental Health (Mannheim, Germany) indicated that calcium levels did not show a significant change from the time of admission to about 1 week later (*n* = 21, Mean_1_ = 2.393, Mean_2_ = 2.391, *t* (20) = 0.075, *p* = 0.941), which roughly corresponds to the time frame between calcium measurement and fMRI session observed in the current study. Additionally, correcting for different time periods between calcium measurement and fMRI sessions in the multiple regression models, used to analyse the fMRI data, did not change the significance of the observed results. This supports the robustness of the presented results. Furthermore, we performed additional partial correlations between calcium scores and craving values, controlling for ADS scores and cumulative ethanol consumption during the last 90 days. The significance of the association between calcium levels and OCDS scores remained unchanged (*r* = − 0.185, *p*_uncorr_ = 0.046). Additionally, we performed univariate analyses of covariance with sex and smoking status as fixed factors and ADS scores, cumulative amount of alcohol consumption during the last 90 days, and time between calcium measurement and fMRI as covariates. Results show that neither of the variables had a significant impact on the magnitude of calcium levels in the current sample (*F* <  = 0.508, *p* = 0.477). This indicates that individual differences on the aforementioned variables did not seem to substantially impact on the results derived from the current dataset. We cannot rule out the possibility that a low calcium concentration is a biomarker for heavier dependent patients, but the missing association between calcium levels and ADS scores does not favor this alternative explanation. Longitudinal serial investigations of plasma calcium levels in future studies are needed to further disentangle the pathophysiological mechanisms underlying the effects of calcium in alcohol-dependent patients. It should be noted that the current dataset did not include a healthy control sample. Hence, we could not determine whether the associations between calcium levels and craving and brain response show differences in healthy participants and clinical samples. This limits the generalizability of presented results. Future studies are needed to differentiate calcium-related effects in healthy controls and dependent patients.

## Conclusion

Our findings show that calcium levels are positively associated with activation in brain areas that are engaged in craving inhibition and negatively associated with subjective alcohol craving. Our data are compatible with the notion that the association between calcium and alcohol craving might be mediated by its effects on alcohol cue-induced brain activation in these brain areas. Further studies are needed to validate the clinical relevance of the overserved findings in alcohol-dependent patients and to further explore the neurobiological basis of the association between plasma calcium levels and alcohol craving.
